# Task-related enhancement in corticomotor excitability during haptic sensing with the contra- or ipsilateral hand in young and senior adults

**DOI:** 10.1186/1471-2202-13-27

**Published:** 2012-03-14

**Authors:** Sabah Master, François Tremblay

**Affiliations:** 1 School of Psychology, University of Ottawa, Vanier Hall, 136 Jean Jacques Lussier, Ottawa, Ontario, Canada K1N 6N5; 2 School of Rehabilitation Sciences, University of Ottawa, Guindon Hall, 451 Smyth Rd., Ottawa, Ontario, Canada K1H 8M5; 3 Élisabeth Bruyère Research Institute, 75 Bruyère St, Ottawa, Ontario, Canada K1N 5C8

## Abstract

**Background:**

Haptic sensing with the fingers represents a unique class of manipulative actions, engaging motor, somatosensory and associative areas of the cortex while requiring only minimal forces and relatively simple movement patterns. Using transcranial magnetic stimulation (TMS), we investigated task-related changes in motor evoked potential (MEP) amplitude associated with unimanual haptic sensing in two related experiments. In Experiment I, we contrasted changes in the excitability of the hemisphere controlling the task hand in young and old adults under two trial conditions, i.e. when participants either touched a fine grating (*smooth trials*) or touched a coarse grating to detect its groove orientation (*grating trials*). In Experiment II, the same contrast between tasks was performed but with TMS applied over the hemisphere controlling the resting hand, while also addressing hemispheric (right vs. left) and age differences.

**Results:**

In Experiment I, a main effect of *trial type *on MEP amplitude was detected (p = 0.001), MEPs in the task hand being ~50% larger during grating than smooth trials. No interaction with age was detected. Similar results were found for Experiment II, *trial type *having a large effect on MEP amplitude in the resting hand (p < 0.001) owing to selective increase in MEP size (~2.6 times greater) for grating trials. No interactions with age or side (right vs. left) were detected.

**Conclusions:**

Collectively, these results indicate that adding a haptic component to a simple unilateral finger action can elicit robust corticomotor facilitation not only in the working hemisphere but also in the opposite hemisphere. The fact that this facilitation seems well preserved with age, when task difficulty is adjusted, has some potential clinical implications.

## Background

Interhemispheric interactions associated with performance of unimanual actions have been the object of much study in recent years. For instance, functional neuroimaging studies have provided evidence that both task complexity and advancing age are critical factors in leading to extra cortical activation in sensori-motor areas ipsilateral to the task hand when participants executed actions with one hand [e.g., [1,2]]. Further evidence for the involvement of ipsilateral motor cortex during unilateral hand actions has come from transcranial magnetic stimulation (TMS) studies in young adults. However, TMS reports in this regard have produced mixed results with evidence of both facilitation and suppression in the ipsilateral "resting hemisphere" while the opposite hemisphere was engaged during unilateral performance of the contralateral hand [e.g., [3-7]. Whether the "resting" hemisphere is facilitated or inhibited appears to depend on factors such as the nature of the unimanual task, particularly the task demands (e.g. low-force phasic vs. high force tonic pinch, congruence between real and imagined movements of the "resting" and task hands), which are known to affect interactions between hemispheres at the sensori-motor level. In view of these findings in young adults, there is a need to better characterize how task demands influence corticomotor facilitation in both the active and resting hemisphere during unimanual actions, especially in the context of human aging.

In a recent series of experiments, we have investigated with TMS the neurophysiological correlates of motor cortical activation associated with unimanual performance using various forms of haptic sensing tasks [8-11]. Haptic sensing with the fingers represents a unique class of manipulative actions, engaging motor, somatosensory and associative areas of the cortex while requiring only minimal forces and relatively simple movement patterns [12,13]. For instance, pattern recognition at the fingertip typically leads to unilateral small amplitude movements to extract spatial information from contour exploration [14]. While such task requires only minimal effort at the motor execution level, pattern recognition is nevertheless cognitively demanding for contour exploration is a slow serial process relying on tactile working memory to integrate spatial information to allow for recognition [14].

Such a class of actions thus provide a unique window to investigate the influence of task demands on motor facilitation associated with unimanual actions. Indeed, our observations showed that robust corticomotor facilitation could be elicited in hemisphere controlling the task hand when participants sensed pattern with the index finger. The fact that this haptic-related enhancement could be abolished by disengaging tactile attention [9] during finger movements pointed to a centrally mediated top-down effect rather than a simple bottom-up afferent mediated increase in corticospinal excitability. Indeed, attending to tactile inputs in the context of haptic sensing engages several cortical regions in the parietal and frontal lobes, including premotor areas [15,16], and the recruitment of this cortical network was likely critical in leading to enhanced corticomotor excitability in the working hemisphere. Such facilitation could be important in finely modulating the corticospinal drive to allow for optimal detection of tactile features as the finger moves. Similar experiments performed in older adults showed that haptic-related enhancement in excitability was still present but depended upon the individual's capacity to perform fine discrimination at the fingertip [17].

In the present report, we extend our previous observations on haptic-related corticomotor facilitation in two series of related experiments destined to further characterize the influence of task demands associated with pattern recognition in young and older adults. In Experiment I, we asked whether haptic-related enhancement in motor excitability in the working hemisphere would be similar between young and older adults, when task difficulty is adjusted to accommodate for age-related changes in tactile perception. In Experiment II, we asked the same question but with regard to the "resting hemisphere", that is whether engaging one hemisphere and one hand in haptic sensing would influence the excitability of the opposite hemisphere and the resting hand to the same extent in young and older adults. We also asked if such crossed modulation would be different depending on whether the right or left hemisphere is engaged in haptic sensing.

## Results

### Experiment I. Corticospinal excitability in the working hemisphere

#### Task performance

**Figure 1:**
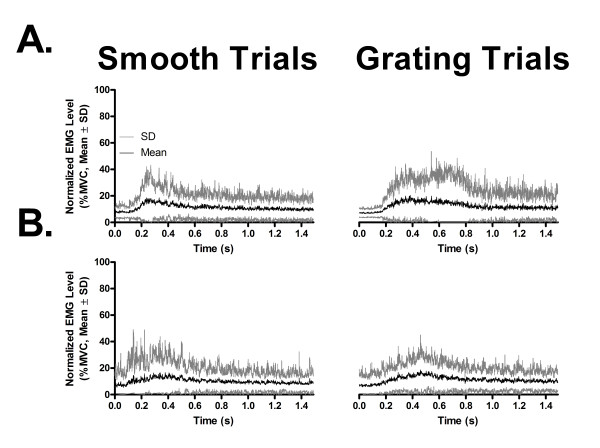
**EMG activity during grating and smooth trials in young and older adults**. Muscle activation pattern elicited in the right FDI in Experiment I during task execution in young (A) and older (B) adults. The traces represent the overall mean (± SD) of all participants' rectified electromyographic (EMG) activity level in each age group (n = 16) recorded prior to application of TMS pulse over the left primary motor cortex, normalized as a percentage of each participant's MVC, for all grating (n = 12) and smooth (n = 12) trials. Note the close similarity in the pattern of muscular activation between the two types of trials, and in both age groups.

In terms of muscle activation, grating and smooth trials elicited a very similar pattern of activity in the FDI muscle, characterized by a sustained increase in activity as the index finger pressed down against the dome. This pattern of EMG activity was similar for both young and senior adults when normalized as a percent of the individual's maximum voluntary contraction (MVC), as shown in Figure 1A and 1B. The average level of activation corresponded to ~10% of the MVC in the two blocks of trials (smooth, 10.0%; grating, 11.7%). Paired comparison revealed no significant difference between the two blocks of trials on mean rectified EMG activity in the 500 ms period preceding the TMS pulse (paired t-test, t_30 _= 2.0, p > 0.05).

#### Task-specific corticospinal facilitation

**Figure 2:**
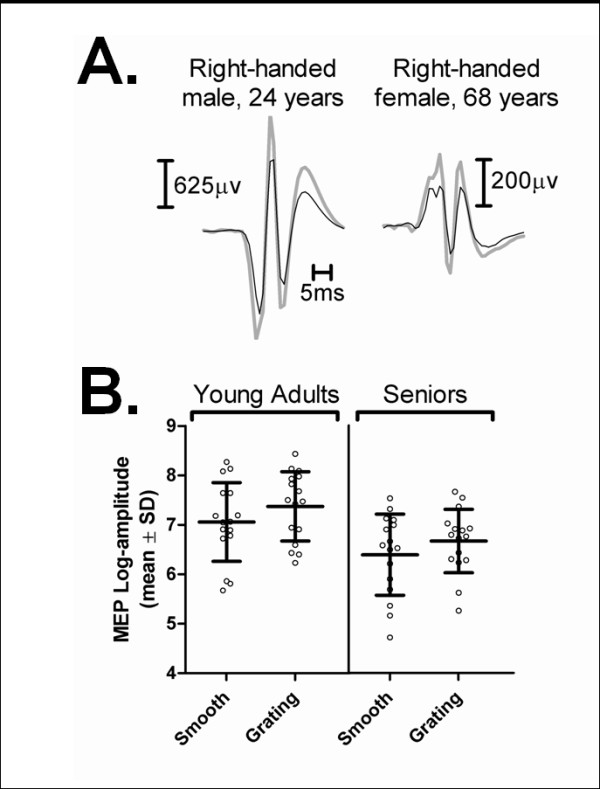
**MEP amplitudes during the smooth vs. grating trials in Experiment I**. A. Examples of task-related differential modulation in MEP amplitude under the two trial types in a typical young (right-handed male, aged 23 years) and a typical older (right-handed female, aged 68 years) participant. Each trace is an average of 12 responses. B. Comparison of mean MEP log-amplitudes in the two trial types (grating/smooth). Each bar represents the mean of individual values computed under each trial type for all young adult (N = 16) and senior participants (N = 16). Note that extra facilitation was observed during the grating trials compared to the smooth trials, and the main effect of trial type was significant for the overall variations in MEP log-amplitude (F_1,30 _= 12.72, p = 0.001, partial eta squared = 0.30).

In general, participants in both age groups exhibited very reliable discrimination in the grating trials with accuracy > 90%, on average. While young participants made slightly more errors (mean, 1.2 ± 0.4) than their senior counterparts (0.3 ± 0.1), the difference was not statistically significant (Mann-Whitney test, p = 0.19). As shown in Figure 2, younger and senior participants showed facilitation of MEP amplitudes during the grating discrimination trials. The main effect of trial type on MEP amplitude was highly significant (F_1,30 _= 12.72, p = 0.001, partial eta squared = 0.30). Age had a main effect on MEP amplitude (F_1,30 _= 7.57, p = 0.01, partial eta squared = 0.20) because seniors generally exhibited smaller MEP sizes than their younger counterparts, irrespective of trial type. No interaction was detected, however, between age and trial type (F_1,30 _= 0.06, p = 0.81), as the two age groups exhibited comparable levels of MEP enhancement in the grating trials (young adults, 46 ± 13%; senior adults, 56 ± 29%). Variations in MEP latency (20.6 ± 0.4 vs. 20.6 ± 0.3 ms, grating and smooth, respectively) and SP duration (101 ± 7.6 vs. 93 ± 6.2 ms, grating and smooth, respectively) were not influenced by trial type (latency, F_1,30 _= 0.06, p = 0.81; silent period, F_1,30 _= 1.76, p = 0.20), age (latency, F_1,30 _= 0.86, p = 0.36; silent period, F_1,30 _= 3.91, p = 0.057), or interactions between trial type and age (F < 2, p > 0.2).

### Experiment II. Corticospinal excitability in the resting hemisphere

#### Task performance

Observations with regard to background EMG level elicited in the task hand were similar to Experiment I, with no significant difference in average levels being detected between smooth and grating trials for both the right hand (smooth, 10.6%, grating, 12.0%, t_31 _= 1.5, p = 0.13) and the left hand (smooth, 10.8%, grating, 11.7%, t_31 _= 1.3, p = 0.19). In the resting hand, mean EMG activity in the resting hand was equivalent to background noise (i.e., ~2% of MVC) for all trials for both the right and left sides in young (2.1%) and older (2.0%) adults.

#### Task-specific corticospinal facilitation

**Figure 3:**
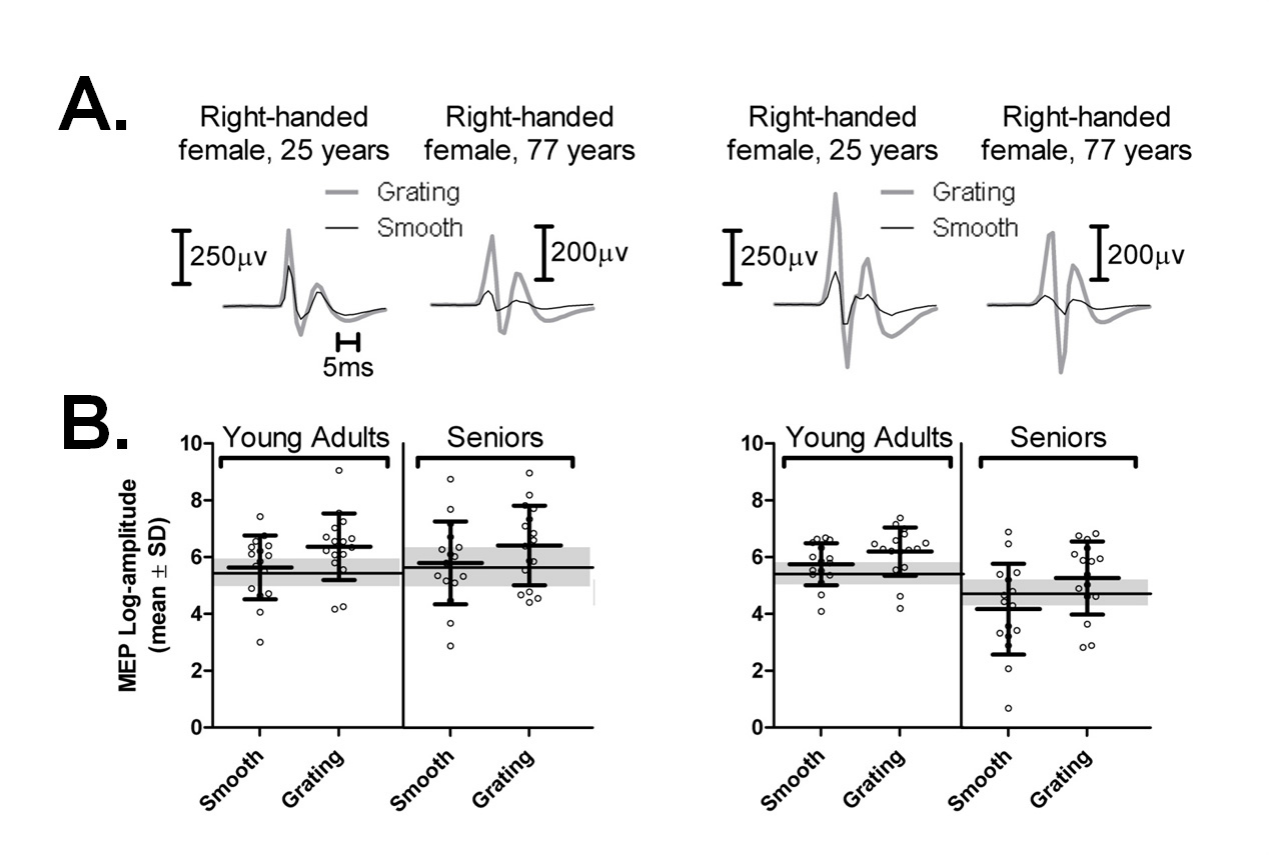
**MEP amplitudes during the smooth vs. grating trials in Experiment II**. A. Examples of task-related differential modulation in MEP amplitude under the two trial types in the left and right resting hands in typical young (right-handed female, aged 25 years) and older (right-handed female, aged 77 years) participants. Each trace is an average of 12 responses. B. Comparison of mean MEP log-amplitudes in the resting hand when the task hand was performing the two trial types (grating/smooth). The gray area represents the mean and standard error of the baseline resting MEP log-amplitudes in the young adult and senior age groups. Each bar represents the mean of individual values computed for the resting hand when the task hand performed each trial type (grating/smooth) for all young adult (N = 16) and senior participants (N = 16). Note the main effect of trial type in the task hand was significant for the overall variations in MEP log-amplitude in the resting hand (F_1,30 _= 16.98, p < 0.001, partial eta squared = 0.36), reflecting enhanced MEP amplitudes in the resting hand when the other hand was doing the grating trials vs. the smooth trials (Bonferroni's p = 0.001) or resting baseline values (Bonferroni's p < 0.001), which were not different from one another (Bonferroni's p = 1.0).

As in Experiment I, participants of both age groups exhibited very reliable and comparable discrimination performance (i.e., > 90%, on average) in the grating trials (overall mean error rate, young, 0.9 ± 0.2; seniors, 0.3 ± 0.1; Mann-Whitney test, p = 0.30). As shown in Figure 3, younger and senior participants showed large MEP facilitation in the resting hand when the other hand was discriminating the grating orientation (F_1,30 _= 16.98, p < 0.001, partial eta squared = 0.36). MEPs in the resting hand were, on average, 2.6 times larger during grating discrimination trials than either during smooth trials or at baseline (Bonferroni's p ≤ 0.001). In fact, MEPs elicited during smooth trials were equivalent in size to those measured at baseline in the resting state (Bonferronni's p = 1.0). As for Experiment I, there was a main effect of age on MEP amplitude in the resting hand (F_1,30 _= 19.18, p < 0.001 partial eta squared = 0.39), owing to the age-related difference in MEP size. There was no interaction between trial type and age in the resting hand (F_1,30 _= 0.37, p = 0.55), seniors showing as much grating facilitation on average (right hand, 183 ± 30%; left hand, 288 ± 77%) as young adults (right hand, 202 ± 25%; left hand, 362 ± 166%). Hemispheric side (F_1,30 _= 0.74, p = 0.40) had no influence on MEP amplitude and showed no interactions with trial type or age (F < 1, p > 0.3). Variations in MEP latency were not influenced by trial type (F_1,29 _= 0.01, p = 0.95), age (F_1,29 _= 2.46, p = 0.13), hand (F_1,29 _= 1.06, p = 0.31), or interactions between any of these factors (F_1,29 _< 4, p > 0.05).

## Discussion

In the present study, we examined variations in corticomotor excitability associated with unimanual pattern recognition at the fingertip in young and older adults. In Experiment I, we showed that haptic enhancement in corticomotor excitability in the working hemisphere was similar in magnitude in young and older adults, when task difficulty was adjusted for age difference. In Experiment II, we showed that haptic enhancement in corticomotor excitability could also be reliably detected in the "resting" hemisphere opposite to the working hemisphere and that this cross-modulation was similar in magnitude between the two age groups and regardless of which hemisphere (i.e., right or left) was engaged in haptic sensing. We will first address the significance of the haptic enhancement observed in Experiment I in the context of aging. Then, we will examine issues pertaining to bilateral corticomotor facilitation as assessed by TMS, in the context of aging with an emphasis on the importance of task demands, in line with the haptic enhancement observed in Experiment II in the "resting" hemisphere.

### Experiment I. Corticospinal excitability in the working hemisphere

In our previous investigation into task-related corticomotor facilitation [17], we found that MEP enhancement with haptic sensing was highly variable in seniors, a variability that linked with the participant's ability to reliably perform haptic discriminations when the task consisted of identifying raised patterns by active stroking of the index finger at a prescribed speed. In this previous study, the inherent difficulty associated with pattern recognition was further complicated by the fact that participants had to externally pace their stroking movement to an auditory tone; hence the possibility of interference with the primary tactile task. In the present study, we show that haptic-related enhancement in corticomotor excitability can be easily elicited in healthy seniors when task demands associated with haptic sensing are reduced both at the kinematics level (i.e., from stroking to simple pressing action) and at the perceptual level (i.e., from patterns to simple grating dimensions). In fact, senior exhibited as much task-related facilitation as young adults when performing grating orientation discriminations. Simplifying task demands in the context of inherently difficult pattern recognition tasks was likely critical in allowing seniors to allocate attentional resources towards tactile inputs coming from the fingertip during grating trials. This observation in seniors, along with the fact that the MEP enhancement was specific for grating trials, reinforces our previous suggestion [9,17] that the likely source of this corticomotor facilitation resides in the activation of the parieto-frontal network subserving tactile attention and tactile processing during haptic sensing. These observations, altogether, indicate that controlling for task difficulty and demand represents a crucial issue when working with older adults in interventions destined to improve fine dexterity.

### Experiment II. Corticospinal excitability in the resting hemisphere

As stressed before, increases in ipsilateral sensorimotor activity have been observed at different levels in the motor system (i.e. peripheral and central) during a variety of unimanual tasks [18-20]. In the present study, we observed that engaging one hemisphere and one hand in haptic sensing was associated with enhanced excitability of the opposite hemisphere associated with the resting hand. In addition, this enhancement was comparable in magnitude between young and old participants, regardless of which hemisphere was engaged in the task. In this regard, our observations are consistent with many TMS reports in young adults, wherein enhanced corticomotor excitability on the "resting" hemisphere has been described when the other was engaged in various unimanual tasks [4,19,21-25]. The present findings may also be interpreted in light of neuroimaging data showing increases in the activity of the ipsilateral sensorimotor cortex during various unimanual tasks, particularly more complex tasks [1,26]. This raises the question as to why smooth trials, which produced similar pattern of background EMG as that seen with grating trials in the task hand, failed to elicit any detectable changes in MEP amplitude in the resting hand; even when compared to baseline values in the resting state. One possible reason for this may reside in the nature of the finger movement, which required only minimal effort on the part of the participant and was simple and highly predictable from trial to trial. In line with this, Tinazzi and Zanette [4] observed a gradual decrease in corticomotor facilitation in the resting hand when the task performed with the opposite hand became more automatic after sequential training. Evidently, grating trials were not susceptible to such training effects with repetitions, because participants were required to deploy tactile attention and use cognitive resources at each trial to discriminate the grating orientation.

Concerning the neural mechanism for the observed haptic enhancement in the "resting" hemisphere, as indicated before, the selectivity of the effect for grating trials points to the parietal-frontal network recruited during tactile attention associated with haptic sensing [15,16] as the primary source of facilitatory influences. In principle, such central facilitation could be exerted at two sites, i.e. either indirectly through the active hemisphere or directly through the "resting" hemisphere. With regard to the first possibility, one possible source is descending ipsilateral facilitation coming from the activated motor cortex through uncrossed corticospinal projections reaching spinal motoneurons. Such a possibility is very unlikely, however, given that ipsilateral corticospinal projections destined to distal hand muscles are rare in primates [27], and were shown recently to have only very weak effects in modulating ipsilateral motoneuronal activity during hand movements [28]. Another related source is irradiation of facilitatory influences or a release from inhibition from the activated primary motor cortex to the homologous cortex through transcallosal projections. Such a possibility is also unlikely, however, for three major reasons. First, most TMS studies looking at interhemispheric influences between motor cortices have emphasized the inhibitory nature of these interactions [see [29] for review] such that conditioning stimuli applied to one hemisphere lead to suppression of MEPs elicited from the opposite hemisphere over a wide range of inter-stimulus intervals. Interhemispheric facilitation can also be elicited but the phenomena has been described as less reliable and remains highly susceptible to variations in experimental paradigm [e.g., [30]. Second, the fact that no mirror activity was detected in the resting FDI homologous to the one engaged in haptic sensing, and that smooth trials elicited no sign of MEP facilitation, argue against the notion of a simple spread of excitation from one motor cortex to the other as the primary source for the observed corticomotor facilitation. Finally, both anatomical tracer work in non-human primates [31] and tractography analyses of diffusion tensor imaging in humans [32] converge to show that primary motor and somatosensory cortices have relatively scant callosal connections when compared to secondary motor or somatosensory areas. This leaves the "resting" hemisphere ipsilateral to the task hand as the most probable site of origin for the observed MEP facilitation in the resting hand. In this respect, a likely source of ipsilateral facilitation during trials with grating discrimination is the recruitment of secondary motor areas, such as the pre-SMA, SMA proper and ventral premotor cortex; which all have been shown to be activated to some degree in tasks involving haptic sensing with the finger [15,16]. As stressed earlier, these secondary motor areas have dense callosal connections and are also densely interconnected with M1 intra-cortically [31,33]. In addition, both SMA and premotor neurons are known to exhibit pre-movement activity during ipsilateral unimanual movement, which contrasts to MI neurons where such premovement activity is seldom found [34,35]. Therefore, bilateral recruitment of secondary motor areas associated with cognitive demand and tactile attention in the context of unilateral haptic sensing seems the most likely source of facilitation to raise motor excitability in the "resting" hemisphere. Yet, one can ask as to why a large increase in corticomotor excitability in the resting hemisphere was not accompanied by overt motor overflow in the resting hand. In this respect, it is possible that interhemispheric inhibitory influences from the active hemisphere to the "resting" hemisphere likely balanced out the facilitation mediated by the secondary motor areas recruited during haptic sensing, reducing it to a sub-threshold level so as to prevent overt mirror movements of the non task hand [36]. To summarize, although we cannot totally rule out the possibility that irradiation from the active hemisphere contributed to the observed ispsilateral MEP enhancement, both anatomical and physiological evidence point to the "resting" hemisphere as the most likely site for the facilitation in link with the recruitment of secondary motor areas in the context of haptic processing.

In line with a previous TMS study comparing right-left differences in the excitability of corticomotor projections to the resting hand when the other hand performed simple movements of varying intensities [37], no difference was found between the two hemispheres with regard to task-related enhancement in corticomotor excitability. However, ipsilateral cortical activity during unimanual performance has often been reported to be more pronounced in the left hemisphere and more important for left hand task performance, particularly for complex tasks [e.g., [1,38]. The present observation that haptic-related corticomotor facilitation in the resting hemisphere was unaffected by hand could be related to the nature of our task, as we have argued before. The fact that our finger task consisted of a simple action requiring minimal effort at the muscular level could have contributed to accentuate differences arising from central influences at the motor cortical level between smooth and grating trials; leading to similar facilitation irrespective of the hand performing the task. Similar to observation between hemispheres, no differences was also observed between young and old participants in the level of MEP facilitation elicited in the "resting" hemisphere when the opposite one was engaged in haptic sensing. This is somewhat in contrast to previous neuroimaging and behavioural studies reporting age-dependant increase in bilateral sensorimotor activation and in peripheral muscle activity during simple unimanual tasks [20,39-41]. Seniors also exhibited less interhemispheric motor inhibition than young adults during performance of unimanual tasks, suggesting that attending to a task may affect inhibitory modulation of ipsilateral motor cortical activity with aging [42]. However, in the present study, the possibility of motor overflow affecting the status of the "resting" hand was greatly reduced by asking participants to refrain from contracting their muscles in the relaxed state and by constant monitoring of EMG level. Such active strategy has been shown to be highly effective in suppressing mirror movements in the non-task hand when complex movements were performed with the other hand in both young and old adults [43]. Thus, any age-dependant increases in motor overflow might have been mitigated by our active control over spreading muscle activity in the resting hand as senior and young participants performed the finger task. Another factor that might have contributed to attenuate age differences is the fact that we controlled for the level of task difficulty. In general, older adults tend to exhibit more widespread cortical activation than younger subjects when motor task demands are increased in terms of complexity [e.g., [2,20,39-41,44]. On this basis, one could have predicted greater haptic-related motor facilitation in the older group owing to the recruitment of a more extensive bilateral frontal and parietal network associated with discrimination of the grating orientation; but this was not the case. As suggested before, such compensatory over-recruitment might have been largely cancelled out in this study by the combined effect of using a simple finger action task and adjusting the level of perceptual difficulty for age for the haptic component. Overall, these observations with regard to the effect of age would be compatible with the compensation-related utilization of neural circuits hypothesis (CRUNCH) theory [45]. This theory suggests that aging results in a loss of hemispheric specialization only in situations in which performance deterioration is evident, to increase compensatory recruitment of available cognitive resources and help improve task performance in seniors [45]. Indeed, poorer unimanual motor task performance in seniors was associated with greater recruitment of ipsilateral M1, decreased callosal size, and integrity, indicating a shift towards excitation in the balance of excitatory and inhibitory interhemispheric interactions [36,39,46]. In the present study, given the careful design of task demands and stimuli to facilitate good performance in seniors in the haptic sensing trials, there would be no reason to expect a compensatory over-recruitment of ipsilateral or contralateral motor areas.

## Conclusions

Finger movements performed in the context of haptic sensing produced a task-specific facilitation in the corticospinal projections destined to intrinsic hand muscles in both senior and young adults, when task demands are adjusted for age. This task-specific facilitation is not only detectable in the working hemisphere controlling the task hand but also in the opposite hemisphere associated with the resting hand. Such results highlight the importance of cognitive factors and task demands in modulating corticospinal output during performance of unimanual actions. These results might have implications in the design of rehabilitative interventions in aging populations. For instance, one could conceive interventions in stroke patients, where haptic sensing is incorporated into hand retraining to facilitate corticospinal drive to the paretic hand. Along the same line, interventions could be directed also at the less affected hand using more demanding haptic sensing tasks to elicited bilateral corticomotor facilitation aiming at promoting recovery of the affected hand. Future work is required to test various haptic sensing tasks in the context of rehabilitation in stroke patients.

## Methods

The Institutional Review Ethics Board approved the study procedure in accordance with the principles of the Declaration of Helsinki and informed consent was obtained before the experimental session. All assessments were performed in a controlled laboratory environment. Each participant received an honorarium for his or her participation.

### Participants

Sixteen healthy young adults (9 males, 7 females, mean age ± SD, 22 ± 2.5 years) and 16 healthy seniors (8 males, 8 females, mean age ± SD: 68 ± 5.1 yrs) from the Ottawa area were included in the study. The majority of subjects were right-handed (young, 13/16; older, 16/16) according to the Edinburgh Handedness Questionnaire. Prior to the experimental session, all participants completed a medical questionnaire to ensure that there were no contra-indications to TMS and no antecedents of conditions likely to affect their performance in the tests. In addition, all participants were screened for the presence of undiagnosed peripheral neuropathies using a graduated Rydel-Seiffer tuning fork, which has been shown to be a valid and reliable instrument for assessing sensory nerve function in the extremities [47,48]. None showed signs of peripheral neuropathy.

Prior to testing participants were also tested to determine spatial acuity theshold at the tip of the right index finger using the JVP domes (Stoelting Co., Wood Dale, IL, USA). The methods and stimuli have been described in detail elsewhere [49]. Briefly, participants were presented successive blocks of trials (n = 30) starting with a wide grating dome (e.g, 3.5 mm-width) and progressing to finer gratings until performance fell to chance level. Each trial consisted of presenting the grating dome in one of two orthogonal orientations according to a pre-determined pseudo-random sequence. Subjects reported the perceived orientation of the grating (i.e., either along or across the fingertip) according to a two-alternative forced-choice paradigm. The tactile spatial acuity threshold was determined by calculating the grating width corresponding to 75% correct discrimination, using a linear interpolation technique [50]. This assessment confirmed that our sample participants, young (mean ± SEM, 1.24 ± 0.1 mm) and senior (1.89 ± 0.2 mm), exhibited spatial acuity thresholds within the range reported previously for similar groups of healthy subjects [49,51,52].

### General procedure for EMG recording and TMS

The recording techniques and TMS procedure have been reported previously [see [9]]. Briefly, EMG activity was recorded using small auto-adhesive surface electrodes (10 mm diameter, Ag-AgCl) placed over the FDI of the right and left hands. EMG signals were amplified (100-500 μV/div), filtered (bandwidth, 16 Hz to 1 kHz), and digitized at 2 kHz (RMP-6004, Nihon-Kohden Corp.; BNC-2090, National Instrument Corp.).

Magnetic stimulation was delivered with a Magstim 200 (Magstim Co. Dyfed, UK) connected to a figure-eight coil (70 mm loop diameter). To determine the optimal site to evoke MEPs in the contralateral hand muscles, the approximate location of the hand motor area on the left hemisphere was explored in 1 cm steps until reliable MEPs could be evoked in the target muscle (FDI). Following this procedure, the relaxed motor threshold was determined using the method advocated by Mills and Nithi [53]. Starting from supra-threshold intensity, the stimulator's output was gradually decreased in 1% steps until no MEP could be evoked for 10 consecutive stimuli. This TMS intensity corresponded to the lower threshold value. From this point, the intensity was gradually increased until MEP's of at least 50 μV peak-to-peak amplitude could be evoked by ten consecutive stimuli. This latter intensity was recorded as the upper threshold value. The relaxed motor threshold (RMT) was defined for each participant as the median intensity between the upper and lower threshold values. Mean RMTs were lower in the right hand, and in young adults (right hand, 35 ± 8.4; left hand, 38 ± 9.2) compared to seniors (right hand, 45 ± 9.7; left hand, 51 ± 8.8; F). The TMS intensity was then fixed at 110% of motor threshold for the remainder of the experiment.

As in our previous studies [9], prior to formal testing, all participants underwent two series of baseline measurements. First, baseline MEP values were derived for each hemisphere (right and left) by delivering TMS with participants instructed to relax. For each side, 12-15 MEPs were recorded at rest with an interstimulus interval of ~ 5 s. Second, estimates of maximal EMG activity for the FDI were derived for each hand by recording muscle activity produced during maximal voluntary contractions (MVC). This was performed by asking participants to abduct (open) their fingers as hard as they could for the duration of a tone lasting 3000 ms against the resistance provided by one of the experimenters (SM) using both hands to hold all four fingers together. The procedure was repeated three times for each with 5 s rest between contractions.

### Experiment I. Corticospinal excitability in the working hemisphere

**Figure 4:**
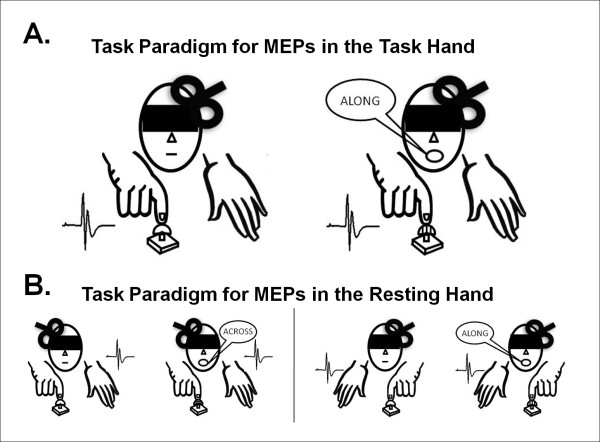
**Task paradigm used to assess corticospinal excitability**. A. *Experiment I*. In both grating and smooth trials, participants were trained to produce a single gentle downwards right index finger movement in sync with the sound of a tone lasting for 1.8 s. During the smooth trials, the finger moved downwards to touch a relatively smooth grating dome (0.5 mm spatial period) with no discrimination requirement. During the grating trials, participants touched a coarse grating dome (1.5 or 3 mm spatial period) and reported whether the grooves ran *along *or *across *the fingertip. In each trial, the TMS pulse was set to trigger over the left motor cortex 1.5 s after the tone, corresponding to the time point in the trial when the finger was touching the dome and the participant was actively discriminating during the grating trials. Contralateral MEPs were recorded in the task hand. B. *Experiment II*. The task conditions were identical to Experiment I except that both the right and left hands were tested in Experiment II, and contralateral MEPs were always recorded in the resting hand instead of the task hand.

In Experiment I, we measured varations in corticomotor excitability in the hemisphere controlling the task hand under two set of conditions which both required participants to gently touch a grating dome (JVP domes, see above) with the distal pad of the index finger. One dome consisted of a fine grating with 0.5 mm groove width, which was designated as the "smooth" dome. This dome was used in *smooth trials*, where participants were simply asked to gently press against the dome without any other requirements. In the second set of trials (*grating trials*), participants performed the same unilateral touching action but this time they were required to report the perceived orientation of the grooves in the presented dome. In grating trials, two different grating dimensions were used for young and senior participants to ensure stimuli would be 150% above mean spatial acuity thresholds in both groups, and thus easily perceptible. The 1.5 mm dome was used in young subjects, whereas the 3.0 mm dome was used in seniors. These grating dimensions were selected on the basis of our estimates of the participants' spatial acuity prior to testing. The orientation of the 1.5 and 3.0 mm grating domes were both reported to be easily detectable by the participants of each age group, though they still required attention. The tasks were always performed with the right index finger and TMS was applied over the left motor cortex to evoke contralateral MEPs in the task hand (see Figure 4A). Participants were first trained to perform smooth trials with the 0.5 mm dome. The task consisted of gently pressing against the dome by flexing the index finger in sync with a tone lasting 1.8 s. Then, participants were trained to perform grating discrimination trials with either the 1.5 (young) or 3.0 mm (senior) dome. The conditions were identical to the smooth trials except that participants were required to attend to the perceived orientation of the grooves as they pressed against the dome. The grating orientation was presented as either *along *or *across *the long axis of the distal finger pad (see, Figure 4A). Immediately at the end of the trials, participants were required to report their responses verbally and were rapidly prompted to respond if no report had been made after the trial completion. Discrimination performance was recorded as the number of correct responses. Participants received appropriate training prior to testing to ensure that contact force and movement characteristics were similar under the two sets of conditions (i.e., smooth trials and grating trials). In both age groups, this training to perform the finger movement in time with the tone and to complete the grating discrimination and report their response only at the end of the trial required 3-5 practice trials (or less than a minute) to complete. The experimenters ensured that participants did not move their finger more during the grating trials than the smooth trials.

Corticospinal excitability was tested with participants comfortably seated in a recording chair and blindfolded. Trials with the smooth and grating domes were presented in two separated blocks of 12 consecutive trials in an order that alternated between participants to control for potential confounders due to variations in attention level, motivation and fatigue [9,17]. In the grating trials, the two orthogonal orientations (across and along) were presented in a pre-determined pseudo-random order with equal probability. In all trials (Figure 4a), TMS was set to trigger towards the end of the task @ 1.5 s in the course of the 1.8 s trial. Pilot testing confirmed that participants were still sensing the grating orientation (as judged by verbal reports) when TMS was delivered at the 1.5 s delay. This delay thus provided an optimal time point to examine task-related MEP facilitation, as participants were actively engaged in sensing for grating orientation. Twelve trials of 1800 ms epochs were recorded under each task condition.

### Experiment II. Corticospinal excitability in the resting hemisphere

In Experiment II, the same task paradigm as in Experiment I was repeated in the same group of participants but this time TMS was applied on the motor cortex of the hemisphere controlling the resting hand (Figure 4B). This experiment allowed for investigation of interhemispheric modulation, as one hemisphere was actively engaged in unimanual performance (i.e., smooth or grating trials) while the opposite hemisphere and opposite hand were at rest. TMS was performed on each resting hemisphere with half the participants being stimulated first on the left (task hand: left) and then on the right (task hand: right), while the other half were tested in the reverse order (i.e., right hemisphere first).

### Analysis of MEP data and background EMG

The details for the procedure for analysis of MEP data and background EMG are given in Master & Tremblay [9]. Briefly, MEP amplitude, latency, silent period (SP), background EMG were measured off-line and averaged to derive mean individual values. For the MVC, EMG signals produced during the last 2000 ms epoch of the MVC were rectified and averaged. This mean rectified maximal EMG value was then used to quantify and compare background EMG level (% MVC) produced during the finger task in Experiment I and II. For this analysis, background EMG levels produced during smooth and grating trials were rectified and averaged using a 500 ms time window preceding the TMS pulse (see Figure 1). Finally, the SP duration was estimated as the interval from MEP onset to the first sign of EMG return.

### Statistical Analysis

MEP amplitudes were not normally distributed (Shapiro-Wilk p < 0.05) and so individual mean values were transformed using the natural logarithm, as suggested by Nielsen [54]. Following this transformation, MEP amplitudes were normally distributed (Shapiro-Wilk p > 0.1). In both Experiment I and II, repeated measures ANOVAs were used to examine the effect of within- and between-subjects factors on each dependent variable. In Experiment I (task hand), the impact of trial type (grating/smooth) and age group (young adults vs. seniors) on the following dependant variables was examined: 1) MEP log-amplitude, 2) MEP latency, and 3) SP duration. In Experiment II (resting hand), baseline was entered into the ANOVA along with smooth and grating trials as repeated factors to contrast variations in MEP amplitude across the three types of trials recorded with the target hand at rest (baseline, smooth, grating). In addition,"hand" was also entered as a repeated factor to examine laterality effects (right vs. left). Age group was the between-subjects factor. The dependent variables were MEP log-amplitude and MEP latency. In both experiments, paired-sample t-tests were used to test for differences on background EMG levels produced between grating and smooth trials. Paired-sample t-tests were also used to test for differences between MEPs recorded in the subset of seniors at 10% of pinch grip and MEPs recorded in these participants during the grating and smooth trials in Experiment I. Performance in the grating task was compared between age groups using the Mann-Whithney U test for performance values were strongly skewed to the left (skewness < -1.0) owing to the low level of task difficulty. All tests were performed using SPSS software version 17.0 for Windows^® ^(Chicago, IL, USA). Figures were prepared using GraphPad Prism version 5.02 for Windows (GraphPad Software, San Diego California USA, http://www.graphpad.com). All values are reported as mean ± SEM.

## Authors' contributions

SM participated in the design of the study, carried out the behavioral testing, performed the statistical analysis and drafted the manuscript. FT conceived of the study, and participated in its design and coordination and drafted the manuscript. Both authors read and approved the manuscript and its revisions.
